# Genomic and Metabolic Characterization of a Potentially Novel *Paenibacillus* Species Isolated as a Laboratory Contaminant Growing on Medium Supporting Cotton Tissue Culture

**DOI:** 10.1002/mbo3.70107

**Published:** 2025-11-06

**Authors:** Ilksen Topcu, Shravan Sharma Parunandi, Tristan Andrew Gregory, LeAnne M. Campbell, Keerti Rathore, Sanjay Antony‐Babu

**Affiliations:** ^1^ Department of Plant Pathology and Microbiology Texas A&M University College Station Texas USA; ^2^ Department of Soil and Crop Sciences Texas A&M University College Station Texas USA

**Keywords:** genome sequence, metabolites, new species, *Paenibacillus* sp, plant tissue culture, taxonomy

## Abstract

*Paenibacillus* sp. *TAB_01*, an isolate recovered from cotton tissue culture plates and a potential novel species within the genus *Paenibacillus* was sequenced using Oxford Nanopore technology. The genome was 7.46 Mb with a G + C content of 52.14%, comprising 7353 total genes, including 6553 coding DNA sequences (CDS) and 159 RNA sequences, including 109 tRNA genes, 46 rRNA genes, and two CRISPR regions. In addition, the genome contains 6553 protein sequences. The results of the digital DNA–DNA hybridization analysis showed that the genome of *Paenibacillus* sp. TAB_01 shared 22.2% similarity with its closest genomic neighbor, *Paenibacillus rigui*. OrthoANIu analysis using USEARCH revealed 78% similarity, while ANI analysis using BLAST indicated 76.8% similarity between the two genomes. The MLSA‐based phylogenetic tree showed that *Paenibacillus* sp. TAB_01 clustered within the *Paenibacillus* genus but formed a separate lineage. Furthermore, *Paenibacillus* sp. TAB_01 exhibited strong plant growth‐promoting potential by producing 112.4 µg/mL of ammonium, 65.6 µg/mL of soluble inorganic phosphate, and 19.5 ng/mL of indole‐3‐acetic acid (IAA). These metabolites were quantified using colorimetric assays with high linearity (*R*² > 0.95). Genomic and metabolic properties indicate that *Paenibacillus* sp. TAB_01 represents a potentially novel species within the genus *Paenibacillus*, clearly distinct from its closest relatives.

## Graphical abstract

This study explores the genomic and metabolic characterization of a potentially novel *Paenibacillus* species isolated from cotton plant tissues. Phylogenetic analysis based on multi‐locus sequence data from six conserved genes reveals that *Paenibacillus* sp. TAB_01 forms a distinct lineage among 19 *Paenibacillus* species. *Paenibacillus* sp. TAB_01 exhibited strong plant growth‐promoting potential, producing 112.4 µg/mL of ammonium, 65.6 µg/mL of soluble inorganic phosphate, and 19.5 ng/mL of indole‐3‐acetic acid (IAA). Genomic and metabolic properties indicate that *Paenibacillus* sp. TAB_01 represents a potentially novel species within the genus *Paenibacillus*, clearly distinct from its closest relatives.

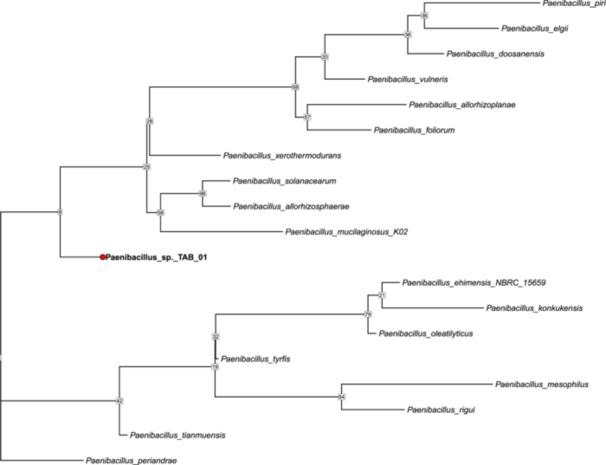

## 1 Introduction

*Paenibacillus* is a genus of facultatively anaerobic, rod‐shaped bacteria that are Gram‐positive or Gram‐variable and capable of forming endospores under aerobic conditions. It was first introduced by Ash et al. (1994) who reclassified it from the *Bacillus* group 3 following detailed analyses of 16S rRNA gene sequences and phenotypic characteristics. Since its reclassification, the taxonomy of *Paenibacillus* has evolved significantly with numerous new species continuously being added. The genus currently consists of 316 species, each with validly published and correctly accepted name (https://www.bacterio.net/genus/paenibacillus, accessed on June 29, 2025). The genus *Paenibacillus* includes bacterial species that play important roles in plants, insects, the environment, and humans (Teng et al. 2003; Ker et al. 2012; Neung et al. 2014; Kämpfer et al. 2022). In this study, we report the whole genome sequencing of *Paenibacillus* sp. TAB_01, a bacterial strain isolated from plant tissue culture medium (found mostly on cotton tissue culture plates, but occasionally observed on media supporting the growth of potato and mung bean cultures), which exhibited characteristics of a potential novel species. In addition, we characterized the production of plant growth‐promoting metabolites by *Paenibacillus* sp. TAB_01.

## 2 Materials and Methods

### 2.1 DNA Extraction and Genome Sequencing, Assembly and Annotation

DNA of the sample was isolated from a bacterial strain recovered from a medium supporting cotton tissue culture, grown on tryptic soy agar supplemented with 50 μg/mL of cycloheximide. DNA extraction was performed using the Zymo Quick Fungal/Bacterial DNA kit (Zymo Research, CA, USA).

The whole genome was sequenced using Oxford Nanopore sequencing. Libraries were prepared using Native Barcoding Kit 24 V14 (product code SQK‐NBD114.24, Oxford Nanopore Technologies, United Kingdom) according to the manufacturer's protocol. Sequencing was performed on a MinION (Oxford Nanopore Technologies, UK) utilizing MinION Flow Cell (R10 version), product code FLO‐MIN114 (Oxford Nanopore Technologies, UK). Base calling was performed using the MinKNOW software in high‐accuracy mode (Oxford Nanopore Technologies PLC, UK), and the quality of the read data was assessed with FastQC v0.12.1 (Andrews 2010). Genome assembly was conducted using Flye v2.9.4 (Kolmogorov et al. 2019), and the resulting contigs were polished with Racon v1.5 (Vaser et al. 2017) using parameters (‐m 8 ‐x ‐6 ‐g ‐8 ‐w 500), followed by Medaka v1.12 (C. Wright and Wykes 2024). Genome assembly quality was evaluated using QUAST v5.0.2 (Gurevich et al. 2013). The genome was annotated using the NCBI Prokaryotic Genome Annotation Pipeline (PGAP). Lastly, secondary metabolite biosynthesis gene clusters were identified using antiSMASH version 7.1.0 (Blin et al. 2023). To assess the genomic similarity between *Paenibacillus* sp. TAB_01 and other *Paenibacillus* species, DNA–DNA hybridization (DDH) values were estimated using the Genome‐to‐Genome Distance Calculator (GGDC), and average nucleotide identity (ANI) was calculated. We selected 18 closely related *Paenibacillus* species from the NCBI database based on the sequence similarity of five conserved protein‐coding genes: *rpoB*, *recA*, *gyrA*, *gyrB*, and *dnaK* to perform comparative genomic analyses (Moore et al. 1987; Goris et al. 2007).

### 2.2 Phylogenetic Tree

Multi‐locus sequence analysis (MLSA) was performed to create a phylogenetic tree for 19 *Paenibacillus* species, including a novel isolate, using six conserved genes: 16S rRNA, *rpoB, recA, gyrA, gyrB*, and *dnaK*. Gene sequences were extracted from GenBank (.gbff) files using a custom BioPython script (Cock et al. 2009). Each gene was aligned with MAFFT v7.526 (Katoh et al. 2019) and concatenated with partitioning information. Maximum likelihood (ML) phylogenetic analysis was performed using RAxML‐NG v1.2.0 with 1000 bootstrap replicates (Kozlov et al. 2019). Phylogenetic tree was visualized in R using the “ape”, ggtree”, and “ggplot2” packages (Wickham 2016; Yu et al. 2017; Paradis and Schliep 2019).

### 2.3 Quantification of Plant Growth‐Promoting Metabolites by *Paenibacillus* sp. TAB_01

#### 2.3.1 Indole‐3‐Acetic Acid (IAA) Production

IAA production was quantified using the colorimetric Salkowski assay (Gordon and Weber 1951; Glickmann and Dessaux 1995). Washed overnight bacterial isolates were cultured in tryptic soy broth (TSB) supplemented with 0.22 µm filtered 2.5 µM l‐tryptophan and incubated at 30  ±  2°C for 72 h with shaking at 150 rpm. After incubation, cultures were centrifuged at 10,000 rpm for 10 min. A 50 µL aliquot of the supernatant was mixed with 150 µL of Salkowski reagent (2.03 g FeCl₃ in a mixture of 17.5 mL of 35% perchloric acid and 32.5 mL distilled water) and incubated in the dark at room temperature for 30 min. The resulting pink coloration was measured at 535 nm using a SpectraMax iD5 Multi‐Mode Microplate Reader (Molecular Devices, San Jose, CA, USA). IAA concentrations were determined using a standard curve generated with pure IAA (0–100 µg/mL).

#### 2.3.2 Nitrogen Fixation Potential

Nitrogen‐fixing ability was evaluated by measuring ammonia accumulation in nitrogen‐free LGI broth medium, as described in Dobereiner et al. (1976) (Dobereiner et al. 1976). The LGI broth medium (pH 6.8  ±  0.2) consisted of: sucrose (20.0 g/L), K₂HPO₄ (0.2 g/L), KH₂PO₄ (0.6 g/L), MgSO₄·7H₂O (0.2 g/L), CaCl₂·2H₂O (0.02 g/L), Na₂MoO₄·2H₂O (0.002 g/L), and FeCl₃·6H₂O (0.01 g/L). Cultures were incubated at 30  ±  2°C for 5 days. Following incubation, cultures were centrifuged at 10,000 rpm for 10 min, after which 50 µL of supernatant was mixed with 50 µL of Nessler's reagent. Samples were incubated at room temperature for 15 min, and absorbance was measured at 435 nm. Ammonium concentration was calculated using a standard curve prepared with ammonium sulfate ((NH₄)₂SO₄) in the range of 0–500 µg/mL.

#### 2.3.3 Phosphate Solubilization

Phosphate solubilization was assessed using the molybdenum blue method (Murphy and Riley 1962), modified for culture supernatants. Bacterial isolates were inoculated into NBRIP broth containing tricalcium phosphate (TCP) as the insoluble phosphate source and incubated at 30 ±  2°C for 5 days. After incubation, cultures were centrifuged at 10,000 rpm for 10 min. For the assay, 10 µL of the supernatant was combined with 20 µL of Solution A (1.25% (NH₄)₆Mo₇O₂₄·4H₂O in 2.5 N H₂SO₄) and 10 µL of Solution B (10% ascorbic acid) and incubated at room temperature for 15 min. Absorbance was measured at 880 nm, and soluble phosphate concentration was determined using a KH₂PO₄ standard curve (0–500 µg/mL).

#### 2.3.4 Carbon Source Utilization Assay

##### 2.3.4.1 Bacterial Inoculum Preparation

*Paenibacillus* sp. TAB_01 was cultured overnight (~18 h) at 27°C in tryptic soy broth (TSB). Following incubation, cells were harvested by centrifugation at 4000*g* for 5 min at room temperature. The resulting pellet was washed twice with sterile distilled water to remove residual nutrients. The washed cells were resuspended with Biolog IF‐0a GN/GP inoculating fluid, a nutrient‐free mineral salt‐based medium recommended for phenotypic microarrays. Cell density was standardized to an optical density (OD₆₀₀) of 0.1 to ensure uniform initial biomass across all wells.

##### 2.3.4.2 Substrate Utilization and Optical Density Measurements

Aliquots of 100 µL from each prepared suspension were dispensed in triplicates into PM1 and PM2 microplates (Biolog Inc., Hayward, CA, USA) containing a total of 190 different carbon substrates. Plates were gently tapped to eliminate air bubbles, sealed with gas‐permeable adhesive film, and incubated under static conditions at 27°C. Optical density readings were taken at 24‐h intervals up to 96 h postinoculation using a SpectraMax iD5 Multi‐Mode Microplate Reader (Molecular Devices, San Jose, CA, USA). Absorbance was recorded at 490 nm (for redox dye reduction) and 590 nm (general biomass indicator).

##### 2.3.4.3 Data Processing and Visualization

Raw optical density values were background‐corrected using the corresponding values from negative control wells. To account for variability across replicates and time points, data were normalized to water and then the baseline (t0) and expressed as the change in absorbance over time (ΔOD). All downstream data processing and statistical analysis were conducted using R (version 4.5.0) (R Core Team 2022). Heatmaps were produced using the “ggplot2” package (Wickham 2016) to visualize differential metabolic activity profiles across substrates and replicates. Substrates showing consistent, time‐dependent increases in OD were considered positively utilized.

#### 2.3.5 Enzymatic Profiling Using API Assay

Enzymatic activity was characterized using the API 20E kit (bioMérieux, France), following the manufacturer's protocol. Bacterial colonies streaked on TSA at 27°C for 18–24 h were suspended in sterile distilled water and centrifuged. The bacterial pellet was resuspended in 5 mL of 0.85% NaCl and vortexed. A 100 µL aliquot of the suspension was inoculated into each well of the API ZYM strip, which contains chromogenic substrates for 20 enzymatic activities. Strips were incubated at 36°C for 24–48 h. Enzymatic activity was subsequently scored according to manufacturer's guidelines.

## 3 Results

### 3.1 Genome Properties and Genomic Comparison

In this study, we performed whole genome sequencing, assembly, and annotation of *Paenibacillus* sp. TAB_01, a bacterial strain recovered during cotton tissue culture, using Oxford Nanopore sequencing. We also conducted phylogenetic tree analysis and characterized the production of plant growth‐promoting metabolites by *Paenibacillus* sp. TAB_01.

Oxford Nanopore Technologies generated a total of 31,263 reads, yielding 265,066,764 bases with an average read length of 1,329,586 bp. Genome assembly produced three contigs with a total length of 7,455,162 bp, a G + C content of 52.14%, and 35.6× coverage. The largest contig was 6,748,452 bp, with an N50 value of 6,748,452 bp, and no gaps. PGAP annotation identified 7,353 total genes, including 6,553 coding DNA sequences (CDS) and 159 RNA sequences. Among the RNA sequences, 109 were tRNA genes and 46 were rRNA genes, comprising 16 5S, 15 16S, and 15 23S rRNA genes. The annotation also identified two CRISPR regions, and 6,553 protein sequences were predicted from the coding regions. In the analysis of secondary metabolite biosynthesis gene clusters, the genome was predicted to encode T3PKS (Type III polyketide synthases), ectoine, terpene, opine‐like metallophore, and lasso peptide (paeninodin).

In the annotation results, the source organism was identified as *Paenibacillus* sp. at the genus level, but not at the species level. To differentiate closely related species within the genome, we selected well‐conserved protein‐coding genes, including *rpoB recA*, *gyrA*, *gyrB* and *dnaK*. The amino acid sequence identities of each protein‐coding gene were analyzed using the NCBI Protein BLAST (Basic Local Alignment Search Tool) at https://blast.ncbi.nlm.nih.gov/Blast.cgi to identify the most similar species sequences available in the NCBI database. Based on protein‐coding gene analysis, 18 closely related *Paenibacillus* species were selected from the NCBI database for DNA–DNA hybridization (DDH) with the Genome‐to‐Genome Distance (GGD) calculator and average nucleotide identity (ANI) calculations (Table 1). Both DDH and ANI are standard methods for assessing overall genomic similarity, providing an estimate of the genetic relatedness between *Paenibacillus* sp. TAB_01 and other *Paenibacillus* species (Moore et al. 1987; Goris et al. 2007). Moreover, multi‐locus sequence analysis (MLSA) based on six conserved genes was performed to construct a phylogenetic tree for 19 *Paenibacillus* species, including the novel isolate. The results showed that *Paenibacillus* sp. TAB_01 is phylogenetically distinct from the other *Paenibacillus* species analyzed.

**Table 1:** Genome information of selected *Paenibacillus* species used in this study for comparisons with the *Paenibacillus* sp. TAB_01 genome.

*Paenibacillus* species	Strain	Genome assembly number	Genome size	G + C (mol%)
*Paenibacillus* sp. TAB_01(This study)	TAB_01		7,453,769	52.14
*Paenibacillus rigui*	JCM 16352	GCA_002234615.1	7,200,000	50.5
*Paenibacillus piri*	MS74	GCA_004354045.1	8,000,000	51
*Paenibacillus mucilaginosus*	K02	GCA_000258535.2	8,800,000	58.5
*Paenibacillus xerothermodurans*	ATCC 27380	GCA_002220865.2	4,400,000	51
*Paenibacillus periandrae*	PM10	GCA_022458865.1	9,800,000	45
*Paenibacillus elgii*	L146	GCA_029625475.1	7,800,000	53.4
*Paenibacillus tyrfis*	MSt1	GCA_000722545.1	8,000,000	53
*Paenibacillus tianmuensis*	CGMCC 1.8946	GCA_900100345.1	6,000,000	52.5
*Paenibacillus foliorum*	LMG 31456	GCA_013141765.1	7,900,000	44.5
*Paenibacillus oleatilyticus*	SM69	GCA_018998565.1	7,900,000	53
*Paenibacillus allorhizosphaerae*	CIP111802	GCA_910594985.1	8,200,000	51
*Paenibacillus mesophilus*	SYSU K30004	GCA_005938385.1	8,800,000	53.3
*Paenibacillus solanacearum*	CIP111600	GCA_910593845.1	7,600,000	54
*Paenibacillus vulneris*	JCM 18268	GCA_039543185.1	7,900,000	49
*Paenibacillus konkukensis*	SK3146	GCA_023523925.1	8,000,000	53.5
*Paenibacillus doosanensis*	CAU 1055	GCA_025060755.1	7,700,000	53.5
*Paenibacillus allorhizoplanae*	CIP 111891	GCA_927798215.1	8,200,000	45
*Paenibacillus ehimensis*	NBRC 15659	GCA_004000785.1	7,500,000	54

DDH estimates were obtained using the GGDC calculator 3.0 (https://ggdc.dsmz.de) with default settings. The local alignment tool was set to MUMMER, optimized for aligning large DNA sequences (Kurtz et al. 2004; Auch et al. 2010; Meier‐Kolthoff et al. 2022). Genomes with a DDH value of 70% or higher are classified as the same species (Auch et al. 2010). The results of the DDH analysis revealed that the *Paenibacillus* sp. TAB_01 genome was 22.2% similar to the closest genomic neighbor, *Paenibacillus rigui*, which was estimated as only 0.02% likely to be the same species.

Average nucleotide identity (ANI) calculations were performed using three different algorithms further supporting the conclusion that *Paenibacillus* sp. TAB_01 represents a novel species. We conducted ANIb (ANI using BLAST), ANIm (ANI using MUMmer), and OrthoANIu (OrthoANI using USEARCH), with a threshold of 95%–96% to distinguish between species (Altschul 1997; Kurtz et al. 2004; Yoon et al. 2017). The JSpecies web server was utilized to calculate ANI based on the BLAST and MUMmer algorithms (Richter et al. 2016), while the EzBioCloud web service was employed to calculate OrthoANIu using USEARCH (http://www.ezbiocloud.net/tools/ani).

**Figure 1:**
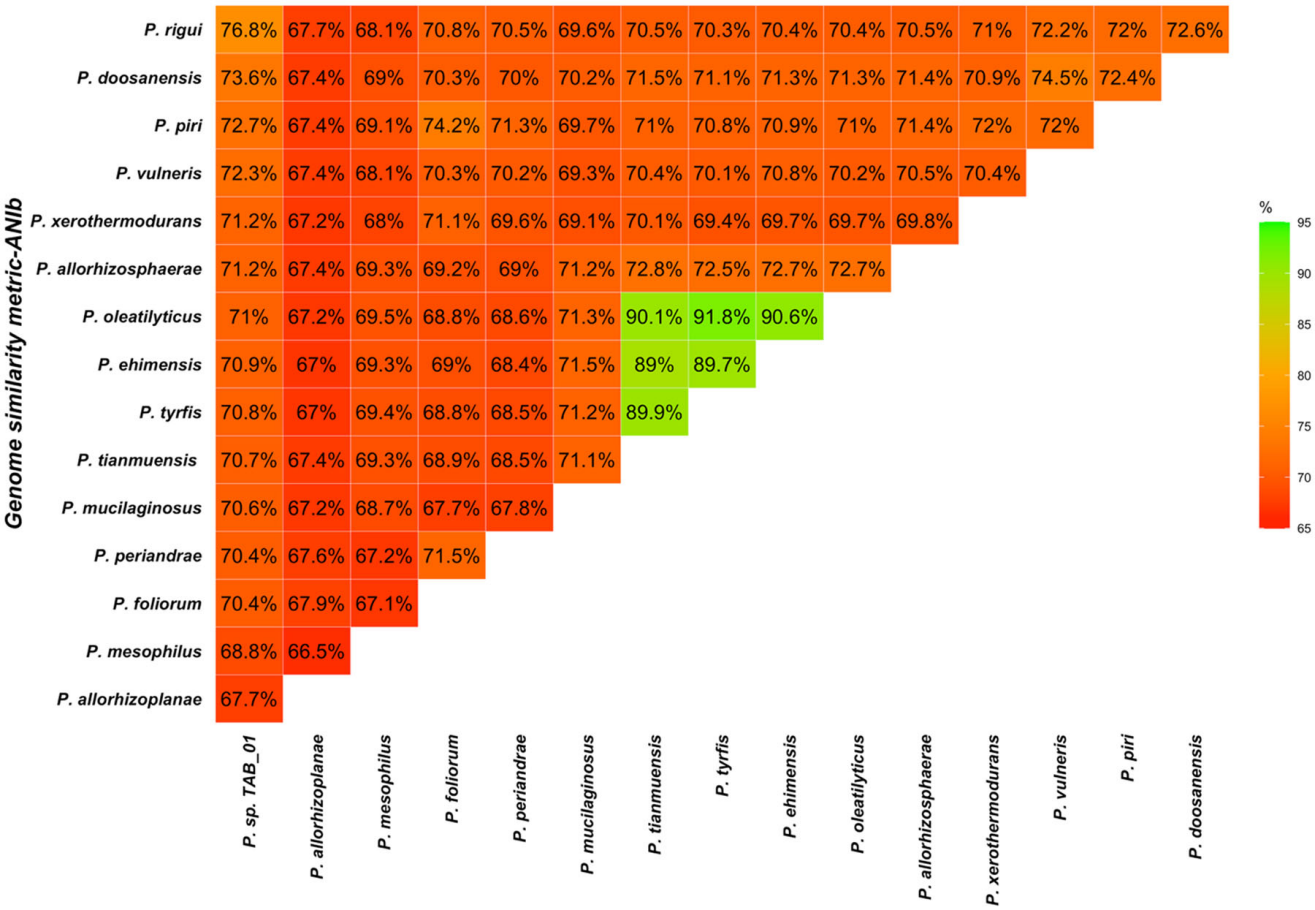
A triangular matrix displaying genome similarity values based on ANI using BLAST. The values within the matrix represent the percentage of similarity from pairwise genomic comparisons between *Paenibacillus* sp. TAB_01 and other *Paenibacillus* species. Higher similarity percentages are indicated by green shading, while lower percentages are represented by red. The species with the lowest similarity to *Paenibacillus* sp. TAB_01 was *Paenibacillus allorhizoplanae* (67.7%), whereas the highest similarity was observed with *Paenibacillus rigui* (76.85%).

We found that genome similarity values using the ANI metric based on BLAST ranged from a minimum of 66.54% to a maximum of 91.84%. The species with the lowest percentage similarity to *Paenibacillus* sp. TAB_01 was *Paenibacillus allorhizoplanae* (67.7%), while the highest percentage similarity was observed with *Paenibacillus rigui* (76.85%) (Figure 1).

**Figure 2:**
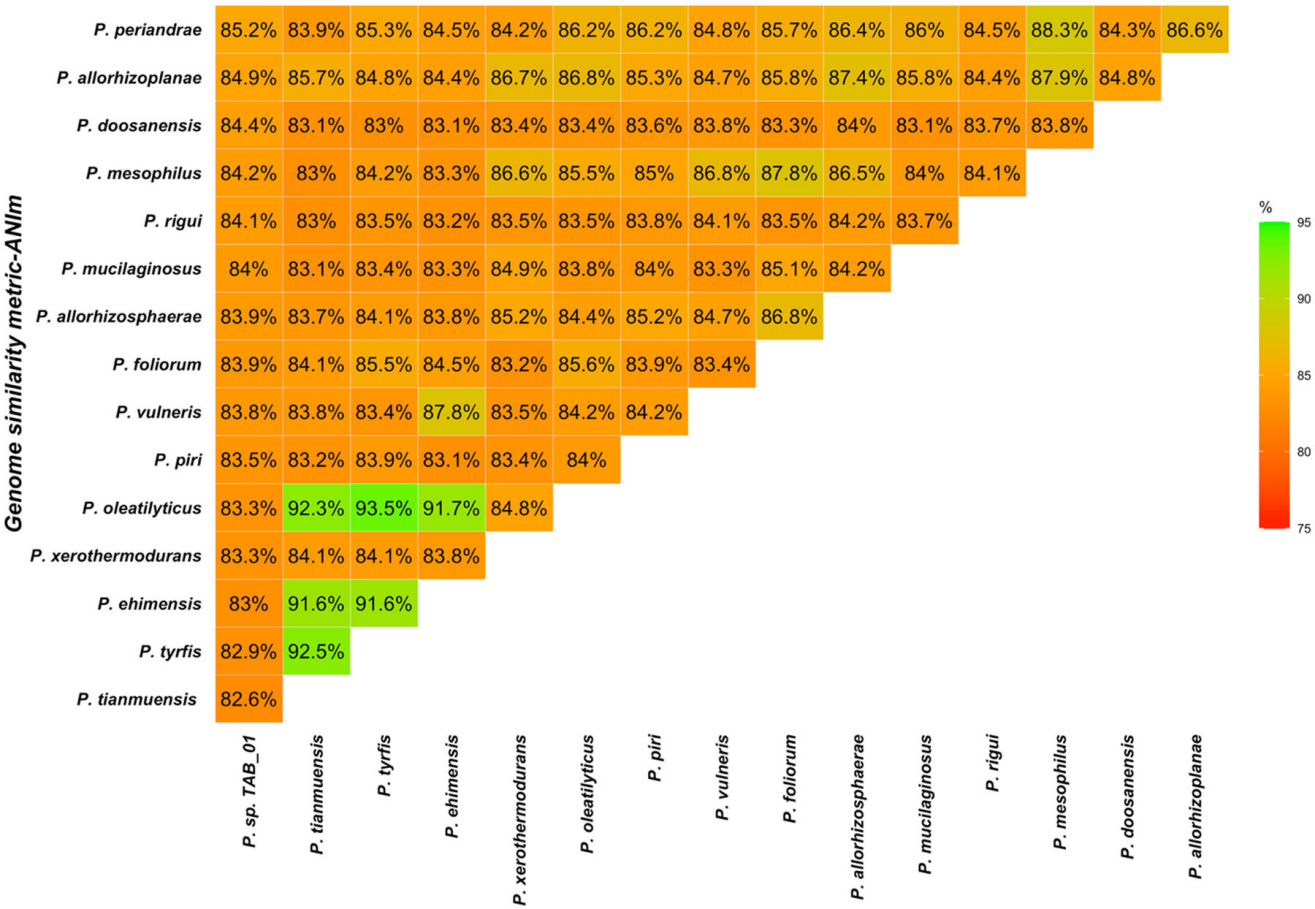
A triangular matrix illustrating genome similarity values based on the ANI metric using MUMmer. The values in the matrix represent the percentage similarity from pairwise genomic comparisons between *Paenibacillus* sp. TAB_01 and other *Paenibacillus* species. Higher similarity percentages are shown in green, while lower percentages are depicted in red. The highest similarity for *Paenibacillus* sp. TAB_01 was 85.21%, observed with *Paenibacillus periandrae*, and the lowest, 82.61%, was found with *Paenibacillus tianmuensis*.

Based on genome similarity values calculated using the ANI metric with MUMmer, the percentage similarity ranged from 82.61% to 93.52%. The highest similarity for *Paenibacillus sp. TAB_01* was 85.21% in comparison to *Paenibacillus periandrae*, while the lowest similarity, 82.61%, was observed with *Paenibacillus tianmuensis* (Figure 2).

**Figure 3:**
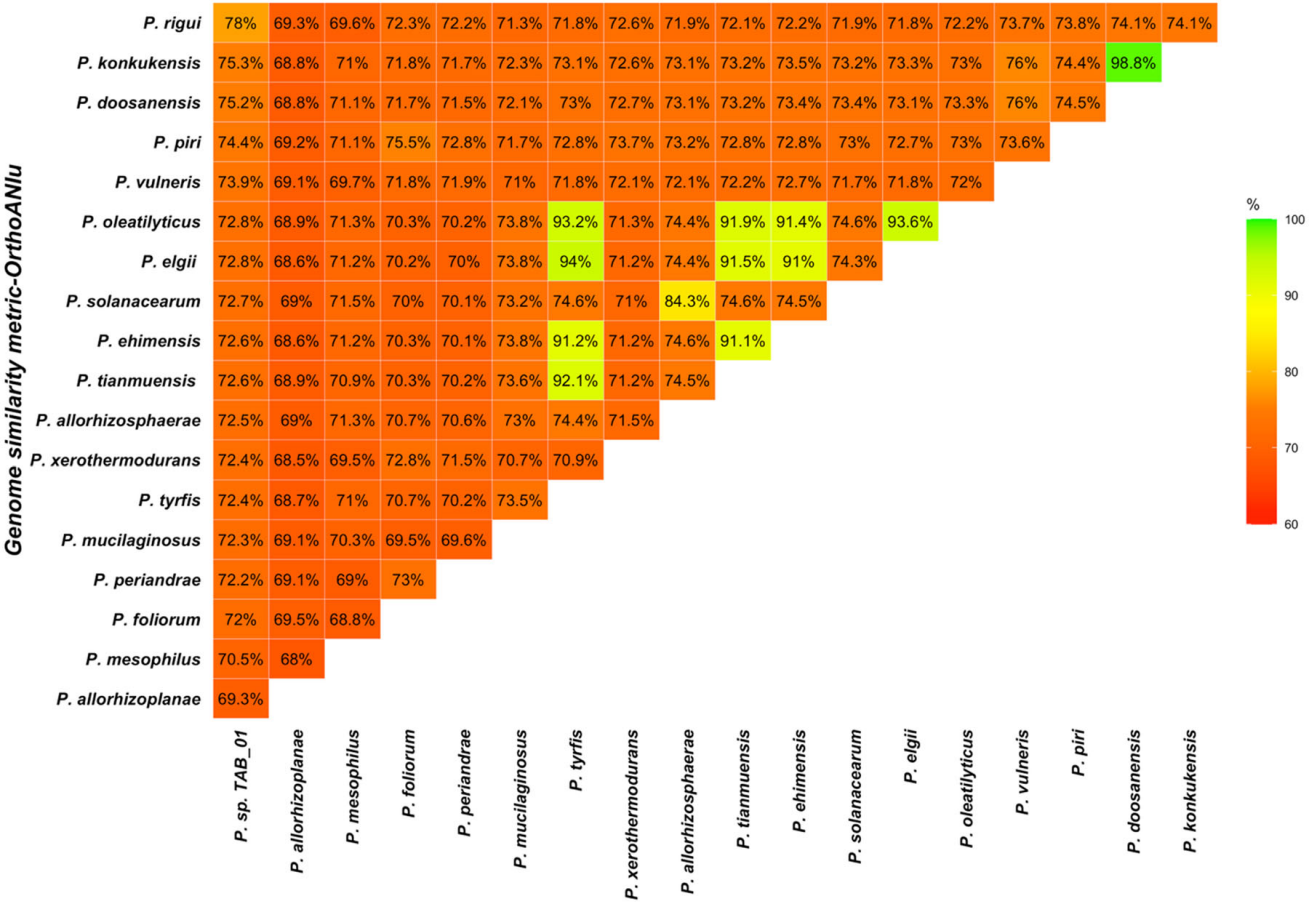
A triangular matrix demonstrating genome similarity values based on OrthoANI using USEARCH. The values in the matrix represent percentage similarities from pairwise genomic comparisons between *Paenibacillus* sp. TAB_01 and other *Paenibacillus* species. The highest similarity percentages are shown in green, while the lowest percentages are depicted in red color. The highest similarity for *Paenibacillus* sp. TAB_01 was 77.97%, observed with *Paenibacillus rigui*, while the lowest similarity, 69.28%, was observed with *Paenibacillus allorhizoplanae*.

Genome similarity values calculated using OrthoANI based on USEARCH ranged from 68.05% to 98.82%. The highest similarity for *Paenibacillus* sp. TAB_01 was 77.97%, observed in comparison to *Paenibacillus rigui*, while the lowest similarity was 69.28% in comparison to *Paenibacillus allorhizoplanae* (Figure 3).

**Figure 4:**
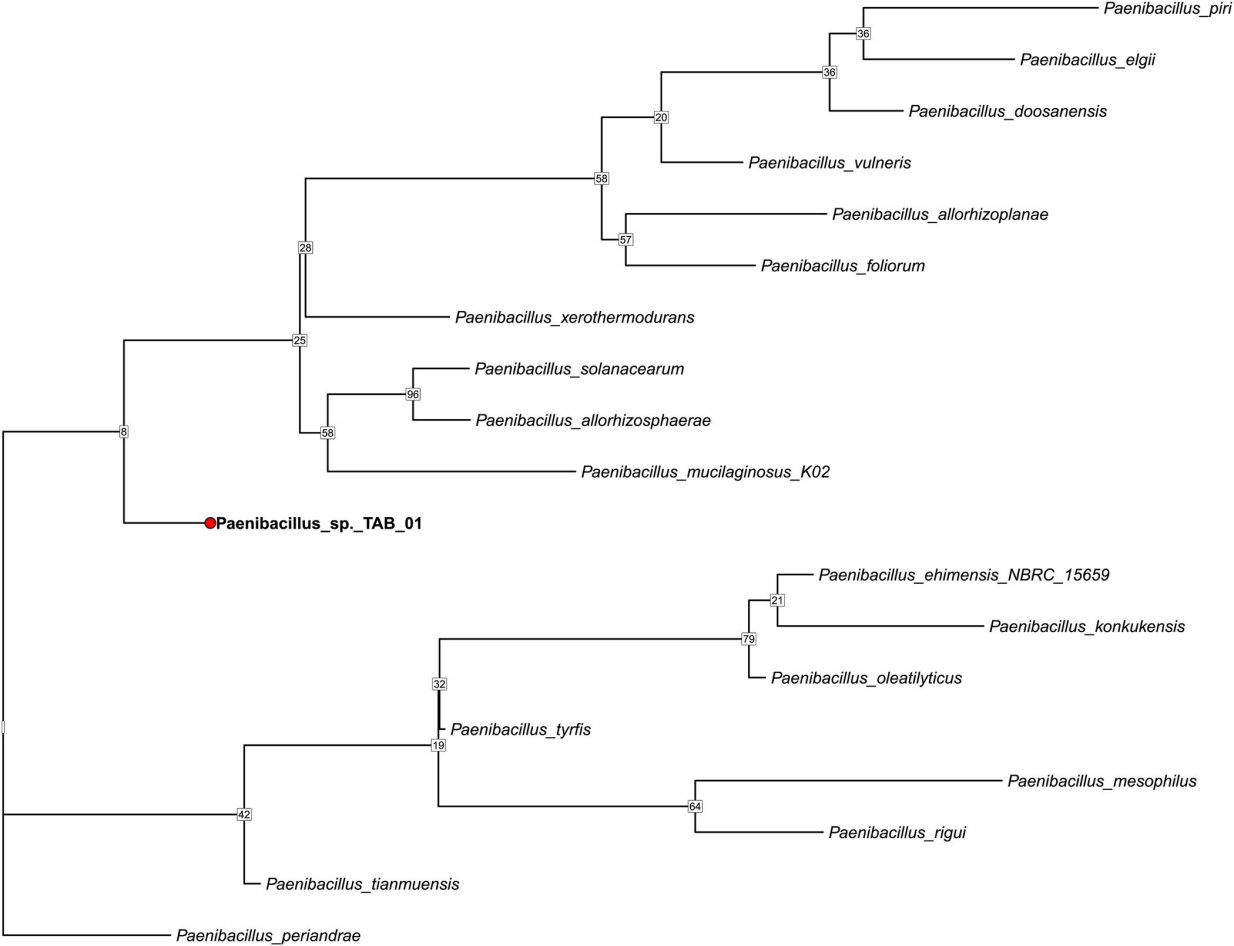
Maximum likelihood (ML) phylogenetic tree based on multi‐locus sequence analysis (MLSA) of 19 *Paenibacillus* species, including a novel isolate identified in this study. The tree was constructed based on six conserved genes, which are 16S rRNA, *rpoB*, recA, gyrA, gyrB, and dnaK. Gene sequences were extracted from GenBank (.gbff) files and aligned with MAFFT v7.526. ML analysis was performed using RAxML‐NG v1.2.0 with 1000 bootstrap.

The maximum likelihood (ML) phylogenetic tree was constructed based on six conserved genes (16S rRNA, *rpoB*, *recA*, *gyrA*, *gyrB*, and *dnaK*) using 1000 bootstrap replicates, which provided strong support for the inferred phylogenetic relationships. This MLSA‐based phylogeny of 19 Paenibacillus species shows that *Paenibacillus* sp. TAB_01 forms a distinct lineage, suggesting it represents a novel species (Figure 4).

### 3.2 Metabolite Profiling of Plant Growth‐Promoting Compounds in *Paenibacillus* sp. TAB_01

#### 3.2.1 IAA, Phosphate, and Nitrogen Fixation Assays

Three colorimetric assays were used to quantify key metabolites produced by *Paenibacillus* sp. TAB_01: indole‐3‐acetic acid (IAA), soluble inorganic phosphate, and ammonium. IAA production was detected using the Salkowski reagent, phosphate solubilization was assessed using the molybdenum blue method, and nitrogen‐fixing capacity was evaluated by measuring ammonium accumulation in nitrogen‐free LGI broth. All assays showed strong linearity within the 100–500 µg/mL range (*R*² > 0.95). Results showed that *Paenibacillus* sp. TAB_01 produced 112.4 µg/mL of ammonium, 65.648 µg/mL of soluble inorganic phosphate, and 19.596 ng/mL of IAA. These results indicate the potential of the strain to promote plant growth via nutrient solubilization and phytohormone production.

#### 3.2.2 Carbon Source Utilization (Biolog PM1 and PM2)

**Figure 5:**
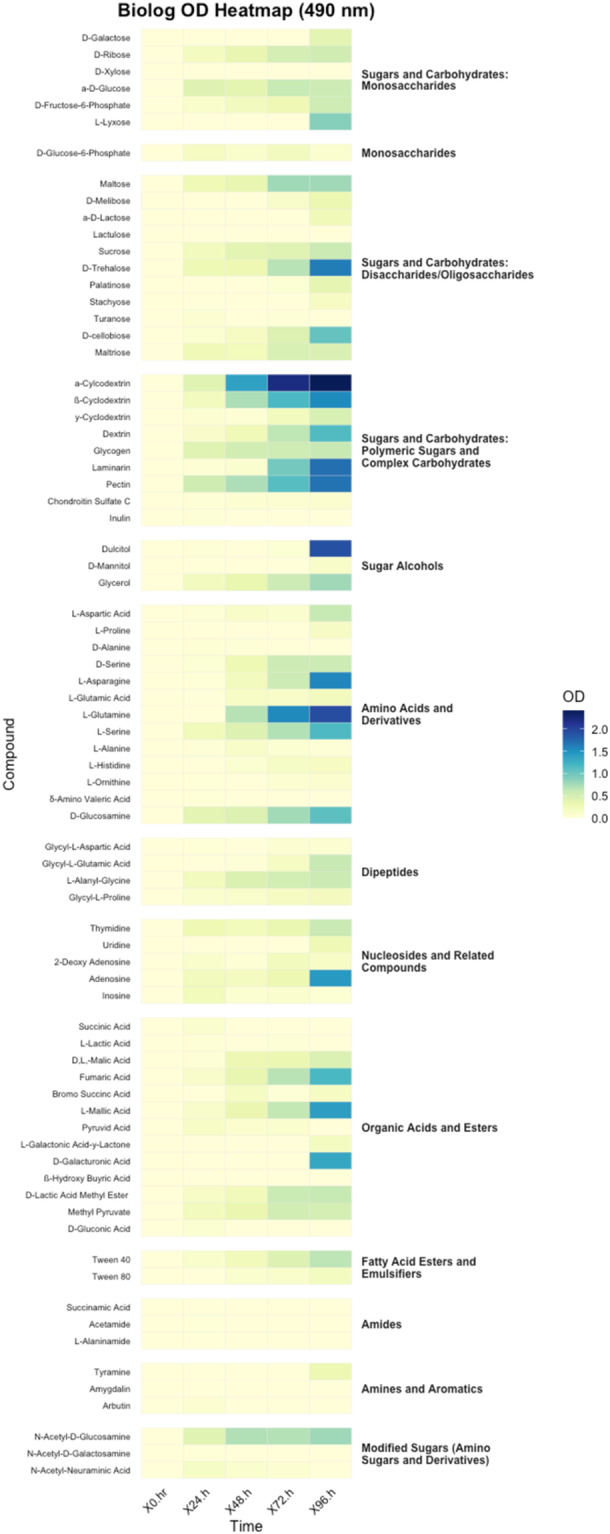
The heatmap shows optical density (OD) values at 490 nm for *Paenibacillus* sp. TAB_01 grown on carbon substrates.

**Figure 6:**
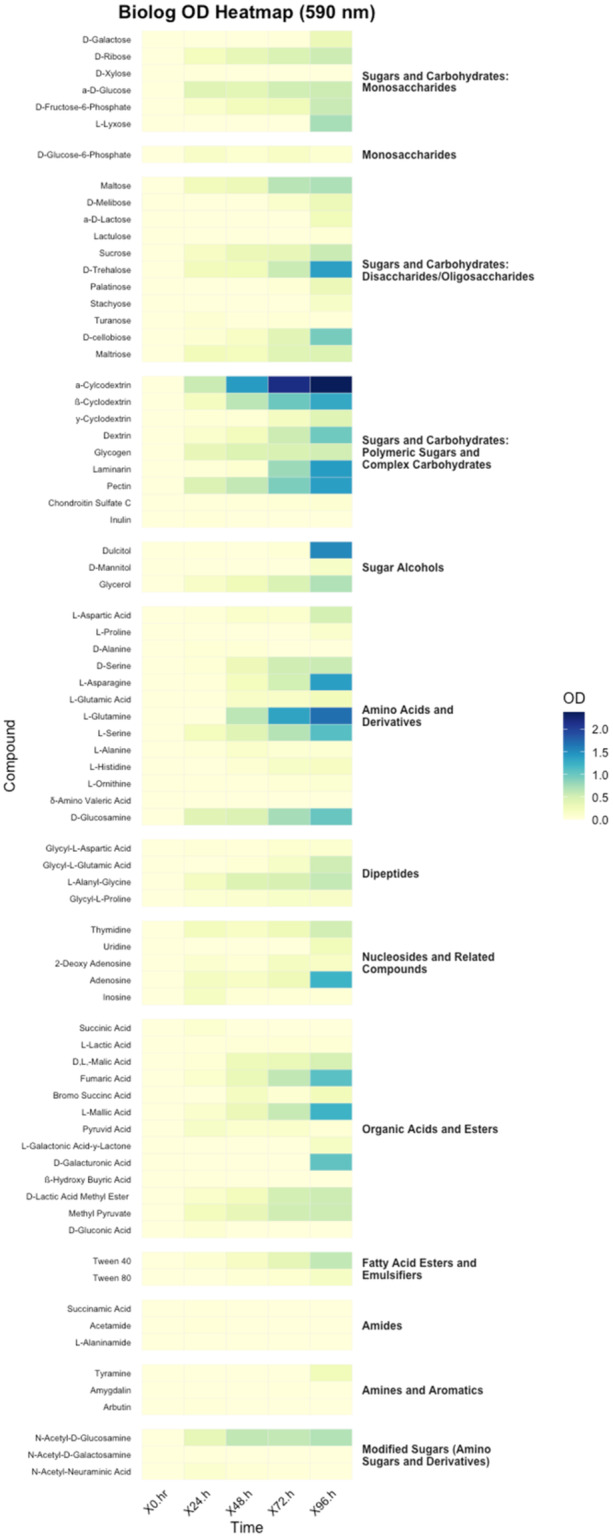
The heatmap shows optical density (OD) values at 590 nm for *Paenibacillus* sp. TAB_01 grown on carbon substrates.

A total of 190 carbon sources, 95 in PM1 and 95 in PM2 were screened to assess the metabolic versatility of *Paenibacillus* TAB_01. Normalized colorimetric readings were calculated as ΔOD =  OD_96h_–OD_0h_. The heatmaps display biochemical classes of carbon substrates based on absorbance readings recorded at 490 nm (redox dye reduction) and 590 nm (general biomass indicator) (Figures 5 and 6). During the heatmap analysis, Gelatin (a protein) showed an exceptionally high level of utilization compared to all other biochemical classes. Its high efficiency made it a significant contributor, but the magnitude of its usage overshadowed the patterns of other components. To allow for better visualization and interpretation of relative trends among the remaining biochemical classes, Gelatin was excluded from the final heatmap. The bacterial isolate exhibited pronounced metabolic activity across a diverse range of substrate classes, as determined by Biolog phenotypic microarrays. Absorbance measurements at 490 and 590 nm were broadly consistent across the tested substrates, indicating parallel trends in metabolic activity and biomass accumulation. However, certain substrates exhibited elevated 490 nm values relative to 590 nm, particularly at later time points. These differences suggest that while metabolic activity remained high, biomass production was limited, possibly due to substrates that support energy generation without contributing substantially to cell growth.

Substrates yielding the highest optical density (OD) values included polymeric sugars and complex carbohydrates (*α‐cyclodextrin*, *β‐cyclodextrin*, *pectin*, *laminarin*, *dextrin*), sugar alcohols (*dulcitol*, *d‐mannitol*), disaccharides (*d‐trehalose*), amino acids (*l‐asparagine*, *l‐glutamine*), amino sugars (*d‐glucosamine*, *N‐acetyl‐d‐glucosamine*), nucleosides (*adenosine*), and organic acids (*d‐galacturonic acid*). OD values increased steadily over 96 h, with a marked response at 590 nm, indicating active substrate metabolism. Among polymeric carbohydrates, utilization of *cyclodextrins*, *laminarin*, *pectin*, and *dextrin* was robust, suggesting enzymatic degradation of β‐1,3‐glucans, galacturonans, and α‐glucans. Sugar alcohols *dulcitol* and *d‐mannitol* also showed elevated oxidation by 72–96 h. Further, disaccharide metabolism was evident with substrates such as *sucrose*, *d‐melibiose*, and *α‐d‐lactose* displaying time‐dependent OD increases, while *maltose*, *lactulose*, and *d‐trehalose* showed moderate but consistent activity. In the amino acid and its derivative category, strong utilization was observed for *l‐glutamine*, *l‐serine*, and *l‐asparagine*. *l‐glutamic acid* and *d‐glucosamine* were also metabolized, indicating integration of both nitrogen and carbon sources. Among organic acids, *l‐malic acid*, *succinic acid*, *pyruvic acid*, and *fumaric acid* were efficiently utilized, while moderate activity was noted for *d‐galacturonic acid* and *β‐hydroxybutyric acid*. In the nucleosides group, *adenosine* and *inosine* supported moderate growth, indicating potential for nucleotide assimilation.

#### 3.2.3 Enzymatic Profiling Using API ZYM Assay

The enzymatic profile of *P. sp. TAB_01* was assessed using the API 20E kit from API ZYM. The isolate exhibited strong activity for arginine dihydrolase, lysine decarboxylase, ornithine decarboxylase, gelatinase, and for glucose and saccharose under fermentation/oxidation conditions, while the remaining enzymes showed low or no activity (Table 2). These results suggest that *P. sp. TAB_01* possesses a selective but functionally relevant enzymatic repertoire, with activity in nitrogen metabolism (via decarboxylases and dihydrolase), proteolysis (gelatinase), and carbohydrate fermentation (glucose and sucrose), which may support its ecological role in soil and plant‐associated environments.

**Table 2:** The table presents the enzymatic profile of *Paenibacillus* sp. TAB_01. Yellow color indicates the recorded results for each corresponding enzyme substrate.

Tests	Active ingredients	Qty (mg/cup.)	Reactions/enzymes	Negative	Positive
ONPG	2‐nitrophenyl‐βD‐galactopyranoside	0.223	β‐galactosidase	Colorless	Yellow (1)
ADH	l‐arginine	1.9	Arginine DiHydrolase	Yellow	Red/orange (2)
LDC	l‐lysine	1.9	Lysine DeCarboxylase	Yellow	Red/orange (2)
ODC	l‐ornithine	1.9	Ornithine DeCarboxylase	Yellow	Red/orange (2)
CIT	Trisodium citrate	0.756	CITtrate utilization	Pale green/yellow	Blue‐green/blue (3)
H2S	Sodium thiosulfate	0.076	H2S production	Colorless/greyish	Red/orange (2)
URE	Urea	0.725	UREase	Yellow	Red/orange (2)
TDA	l‐tryptophane	0.38	Tryptophane DeAminase	Yellow	Reddish brown
IND	l‐tryptophane	0.19	INDole production	Pale green/yellow	Pink
VP	Sodium pyruvate	1.9	Acetoïn production (Voges Proskauer)	Colorless/pale pink	VP 1 + VP 2/10 min
GEL	Gelatin (bovine origin)	1.9	GELatinase	No diffusion	Diffusion of black pigment
GLU	d‐glucose	1.9	Fermentation/oxidation (GLUcose)	Blue/blue‐green	Yellow/greyish yellow
MAN	d‐mannitol	1.9	Fermentation/oxidation (MANnitol)	Blue/blue‐green	Yellow
INO	Inositol	1.9	Fermentation/oxidation (INOsitol)	Blue/blue‐green	Yellow
SOR	d‐sorbitol	1.9	Fermentation/oxidation (SORbitol)	Blue/blue‐green	Yellow
RHA	l‐rhamnose	1.9	Fermentation/oxidation (RHAmnose)	Blue/blue‐green	Yellow
SAC	d‐sucrose	1.9	Fermentation/oxidation (SACcharose)	Blue/blue‐green	Yellow
MEL	d‐melibiose	1.9	Fermentation/oxidation (MELibiose)	Blue/blue‐green	Yellow
AMY	Amygdalin	0.57	Fermentation/oxidation (AMYgdalin)	Blue/blue‐green	Yellow
ARA	l‐arabinose	1.9	Fermentation/oxidation (ARAbinose)	Blue/blue‐green	Yellow

*Note:* For each enzyme, negative and positive outcomes are represented by different colors. Yellow color marked represents the outcome of that particular enzyme assay. For example, in case of ONPG, the reaction did not show any color which was marked in negative column.

## 4 Discussion

In this study, we characterized the genomic properties of *Paenibacillus* sp. TAB_01, a bacterial strain recovered from cotton tissue culture medium, using Oxford Nanopore sequencing, and assessed the production of plant growth‐promoting metabolites by *Paenibacillus* sp. TAB_01. Our results indicated that the test strain, TAB_01, belongs to the genus *Paenibacillus*, with its closest relative being *Paenibacillus rigui*, based on 78% similarity from OrthoANIu using USEARCH and 76.8% similarity from ANI using BLAST. Moreover, *Paenibacillus periandrae* showed 85.2% similarity based on ANIm using MUMmer algorithms. Furthermore, the results of DDH analysis demonstrated that the similarity between *Paenibacillus* sp. TAB_01 and other *Paenibacillus* species was less than 70%. The MLSA‐based maximum likelihood phylogenetic tree suggests that *Paenibacillus* sp. TAB_01 is distantly placed from all currently described *Paenibacillus* species, indicating that it represents a potentially distinct lineage. Comparison of *Paenibacillus* sp. TAB_01 with *Paenibacillus rigui* and *Paenibacillus periandrae* showed that the DNA G + C content of *Paenibacillus* sp. TAB_01 was 52.14 mol%, while *Paenibacillus rigui* had a G + C content of 50.5 mol%, and *Paenibacillus periandrae* had a G + C content of 45 mol%. Moreover, the number of contigs for *Paenibacillus rigui* and *Paenibacillus periandrae* was 106 and 256, respectively, using the Illumina sequencing platform. In contrast, we identified three contigs for *Paenibacillus* sp. TAB_01 using Oxford Nanopore Sequencing. Before the development of Oxford Nanopore, which is a long‐read sequencing platform, Illumina, a short‐read sequencing platform, was commonly used for genomic characterization. Previous studies showed that Oxford Nanopore outperforms Illumina in terms of contig number (Linde et al. 2023; Bejaoui et al. 2025; Dakroub et al. 2025). Bejaoui et al. (Bejaoui et al. 2025) employed Flye, the assembly tool recommended for Oxford Nanopore data, and reported an average of five contigs per genome using Oxford Nanopore, compared to an average of 194 contigs for Illumina based assemblies. Similarly, Dakroub et al. (Dakroub et al. 2025) revealed that Oxford Nanopore produces fewer contigs compared to Illumina, especially when sequencing depth is high. They also noted that Illumina assemblies tend to be more fragmented due to the limitations of short read sequencing.

The CRISPR‐associated genes, known as cas, provide adaptive immunity that protects against viruses, plasmids, and transposable elements and Cas gene clusters are highly diverse (Haft et al. 2005; A. V. Wright et al. 2016; Pourcel et al. 2020). The transcripts produced from the CRISPR array are noncoding and are often very long (Pourcel et al. 2020). We identified two CRISPR arrays in the genome, along with seven associated cas genes. The first CRISPR array is located between positions 4,476,532 and 4,478,093, spanning 1562 base pairs. The second CRISPR array is positioned between 4,486,405 and 4,488,227, with a total length of 1823 base pairs. The cas genes annotated in proximity to these arrays include cas1, cas2, cas3, cas4, cas5, cas7, and cas8, which are collectively involved in spacer acquisition, interference, and CRISPR complex assembly.

The observed substrate utilization profile highlights the metabolic versatility of the isolate, with strong preference for complex carbohydrates and polyols, suggesting adaptation to environments rich in plant‐derived polymers. The pronounced oxidation of *α‐ and β‐cyclodextrins*, along with *laminarin* and *pectin*, supports the presence of a robust suite of carbohydrate‐active enzymes (CAZymes) capable of degrading complex glycosidic linkages, as frequently observed in plant‐associated and soil‐dwelling microbes (Lombard et al. 2014; Berlemont and Martiny 2015). These capabilities likely confer competitive advantage in the rhizosphere, where breakdown of polysaccharides such as pectin and laminarin releases oligomers that serve as valuable carbon sources (Schellenberger et al. 2010).

Efficient metabolism of sugar alcohols such as *dulcitol* and *d‐mannitol*, in combination with disaccharides including *sucrose*, *d‐melibiose*, and *lactose*, indicates an integrated saccharolytic system that enables the bacterium to exploit both storage and structural sugars. These compounds likely feed into central metabolism via glycolysis or the pentose phosphate pathway following hydrolysis by glycosidases, reflecting broad substrate specificity and redundancy typical of saprophytic bacteria (Martens et al. 2011). Moderate activity on *maltose* and *d‐trehalose*, both linked via α‐1,4 and α,α‐1,1 glycosidic bonds, respectively, underscores the isolate's enzymatic versatility in handling diverse linkage types.

Nitrogen metabolism was particularly active, as evidenced by utilization of *l‐glutamine*, *l‐asparagine*, and *l‐serine*, which are among the most readily assimilable amino acids in bacterial systems (Reitzer 2003). The coupled oxidation of *l‐glutamic acid* and *l‐glutamine* suggests involvement of the glutamine synthetase–glutamate synthase (GS‐GOGAT) cycle, a key regulatory hub for ammonium assimilation (Leigh and Dodsworth 2007). The simultaneous metabolism of *d‐glucosamine* and *N‐acetyl‐d‐glucosamine* reflects the organism's capacity to connect amino sugar degradation with glycolytic and peptidoglycan recycling pathways, consistent with roles of these sugars in both carbon acquisition and cell wall turnover.

The isolate's catabolism of TCA cycle intermediates (*malic acid*, *succinic acid*, *pyruvic acid*, *fumaric acid*) aligns with the presence of a fully functional central carbon metabolism, allowing direct assimilation of these compounds for energy production and biosynthesis. Moderate oxidation of *d‐galacturonic acid* and *β‐hydroxybutyric acid* points to auxiliary routes such as the glyoxylate cycle or β‐oxidation, enabling flexibility under carbon‐limited conditions. Finally, utilization of *adenosine* and *inosine* supports the presence of salvage pathways that allow the recycling of purine bases for nucleic acid synthesis, an important trait for survival in oligotrophic environments. Altogether, the isolate exhibits crucial traits of ecological adaptability and trophic versatility, well‐suited for life in nutrient‐variable soil or rhizosphere habitats.

In conclusion, the genomic and metabolic characterization of *Paenibacillus* sp. TAB_01 indicates that it represents a potentially novel species within the genus Paenibacillus, clearly distinct from its closest relatives based on genomic similarity and phylogenetic placement. Its ability to solubilize phosphate, fix nitrogen, and produce IAA further suggests its potential utility in agricultural or environmental applications. Further, *in planta* studies are required to confirm the strain for its bioprospecting potential.

## Author Contributions

**Ilksen Topcu:** writing – original draft, methodology, formal analysis, data curation. **Shravan Sharma Parunandi:** writing – original draft, data curation, formal analysis, methodology. **Tristan Andrew Gregory:** data curation. **LeAnne M. Campbell:** writing – review and editing. **Keerti Rathore:** resources, writing – review and editing. **Sanjay Antony‐Babu:** writing – review and editing, supervision, funding acquisition, investigation, validation.

## Ethics Statement

The authors have nothing to report.

## Conflicts of Interest

The authors declare no conflicts of interest.

## Data Availability

The data that support the findings of this study are openly available in NCBI at https://www.ncbi.nlm.nih.gov/bioproject/PRJNA1168290. Raw data have been deposited in NCBI with accession number PRJNA1168290. The assembled genome and its annotation have been deposited in GenBank under the accession number JBIFNP000000000.
